# Advanced Subclinical Atherosclerosis and Low-Dose Aspirin: Guideline-Based Extension Into Asymptomatic Chronic Coronary Syndromes

**DOI:** 10.1016/j.jacadv.2025.101855

**Published:** 2025-05-29

**Authors:** Michael J. Blaha, Francesca Santilli, Dirk Sibbing, Augusto Lavalle Cobo, Magdy Abdelhamid, Gerhard Albrecht, Li Li

**Affiliations:** a Johns Hopkins Ciccarone Center for the Prevention of Cardiovascular Disease, Baltimore, Maryland, USA; b Department of Medicine and Aging and Center for Advanced Studies and Technology, University of Chieti, Chieti, Italy; c Privatklinik Lauterbacher Mühle am Ostersee, Iffeldorf, Germany; d Department of Cardiology, Klinikum der Universität München, Ludwig-Maximilians-University, Munich, Germany; e Department of Cardiology, Sanatorio Otamendi, Ciudad Autonoma de Buenos Aires, Argentina; f Department of Cardiovascular Medicine, Faculty of Medicine, Kasr Al Ainy, Cairo University, Giza, Egypt; g Medical & Clinical Affairs Consumer Health, Bayer U.S. L.L.C., Whippany, New Jersey, USA; h Medical Affairs & Pharmacovigilance, Pharmaceuticals, Bayer AG, Berlin, Germany

**Keywords:** atherosclerosis, aspirin, asymptomatic chronic coronary syndrome, coronary artery disease

**Figure:**
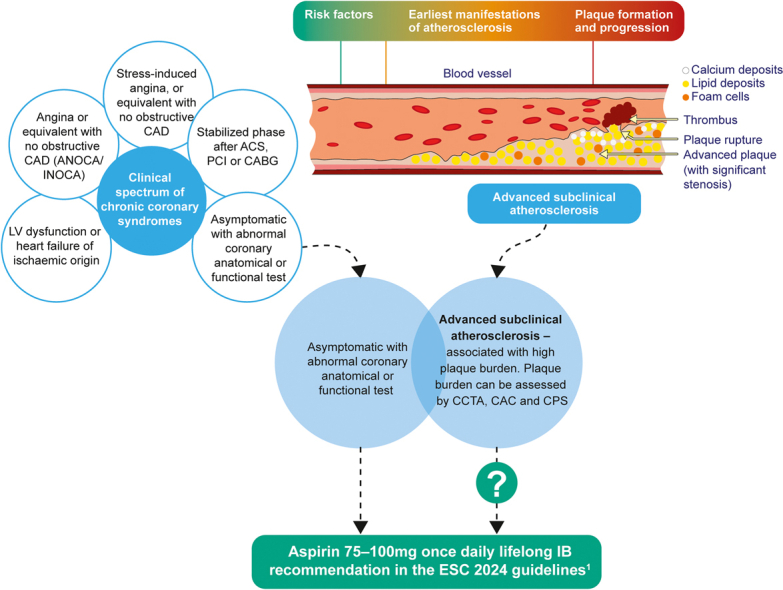

Chronic coronary syndromes (CCSs) are defined as a range of clinical presentations or syndromes that arise due to structural and/or functional alterations related to chronic diseases of the coronary arteries and/or microcirculation.^1^ While chronic coronary diseases are often stable for long periods, they are progressive and may suddenly develop into acute coronary syndromes.

The European Society of Cardiology (ESC) guidelines for the management of CCS have been updated based on changing epidemiology, diagnostic and risk prediction strategies, risk classification, and new evidence in the field for CCS, as well as medical therapy landscapes. One of the notable updates to the guidelines concerns the recommendation for low-dose aspirin in patients without prior myocardial infarction or revascularization but with evidence of significant obstructive coronary artery disease (CAD). This recommendation has been upgraded from class IIb in 2019 to class Ib in 2024.^1^^,^^2^

The key pathological mechanism underlying most epicardial CAD is atherosclerosis, a complex process that develops over many years and even decades before stenosis. The degree of stenosis was traditionally used to assess an individual's cardiovascular (CV) risk and determine whether the patient requires preventive therapy. Obstructive coronary stenoses have typically been defined using visual thresholds of either 50% or 70% stenosis, and stenosis of ≥50% is usually an indication for secondary prevention level therapy.

However, increasing evidence demonstrates that both nonobstructive and obstructive CAD, detected by coronary computed tomography angiography, confer an increased long-term risk of major adverse CV events and mortality.^3^ In fact, atherosclerotic plaque burden, and not stenosis per se, appears to be the main predictor of CV events over a mid-term time horizon.^4^, ^5^, ^6^ Atherosclerotic plaque burden can be determined in a number of ways, including coronary computed tomography angiography, the coronary artery calcium (CAC) score, and carotid ultrasound. Aspirin is of particular interest since in guidelines it generally remains in the “obstructive CAD” paradigm as opposed to the “plaque burden” paradigm. However, we believe that the quantification of plaque burden can help individualize the allocation of aspirin.^7^^,^^8^

A recent publication by our writing group highlighted a novel category within the CV risk continuum—advanced subclinical atherosclerosis.^9^ These asymptomatic patients are at a high risk of CV events due to the presence of substantial underlying subclinical atherosclerosis as determined exclusively by their plaque burden. As such, they are best thought of as distinct from traditional primary prevention (where aspirin has a limited role) and secondary prevention (where aspirin is categorically recommended). For example, patients with CAC >300 have atherosclerotic cardiovascular disease events equivalent to patients with known obstructive CAD, even without exploration for degree of coronary stenosis.^10^

If patients with asymptomatic coronary stenosis of 50% are considered to have CCS and, therefore, benefit from low-dose aspirin, then should these recommendations also apply to those with advanced subclinical atherosclerosis who have been demonstrated to have similar event rates? We think the answer is yes. We believe that the concepts of advanced subclinical atherosclerosis and asymptomatic CCS overlap, and the recent guideline regarding the management of asymptomatic CCS with low-dose aspirin can be reasonably extended to advanced subclinical atherosclerosis when there are no bleeding risk factors (Figure 1).

**Figure 1:**
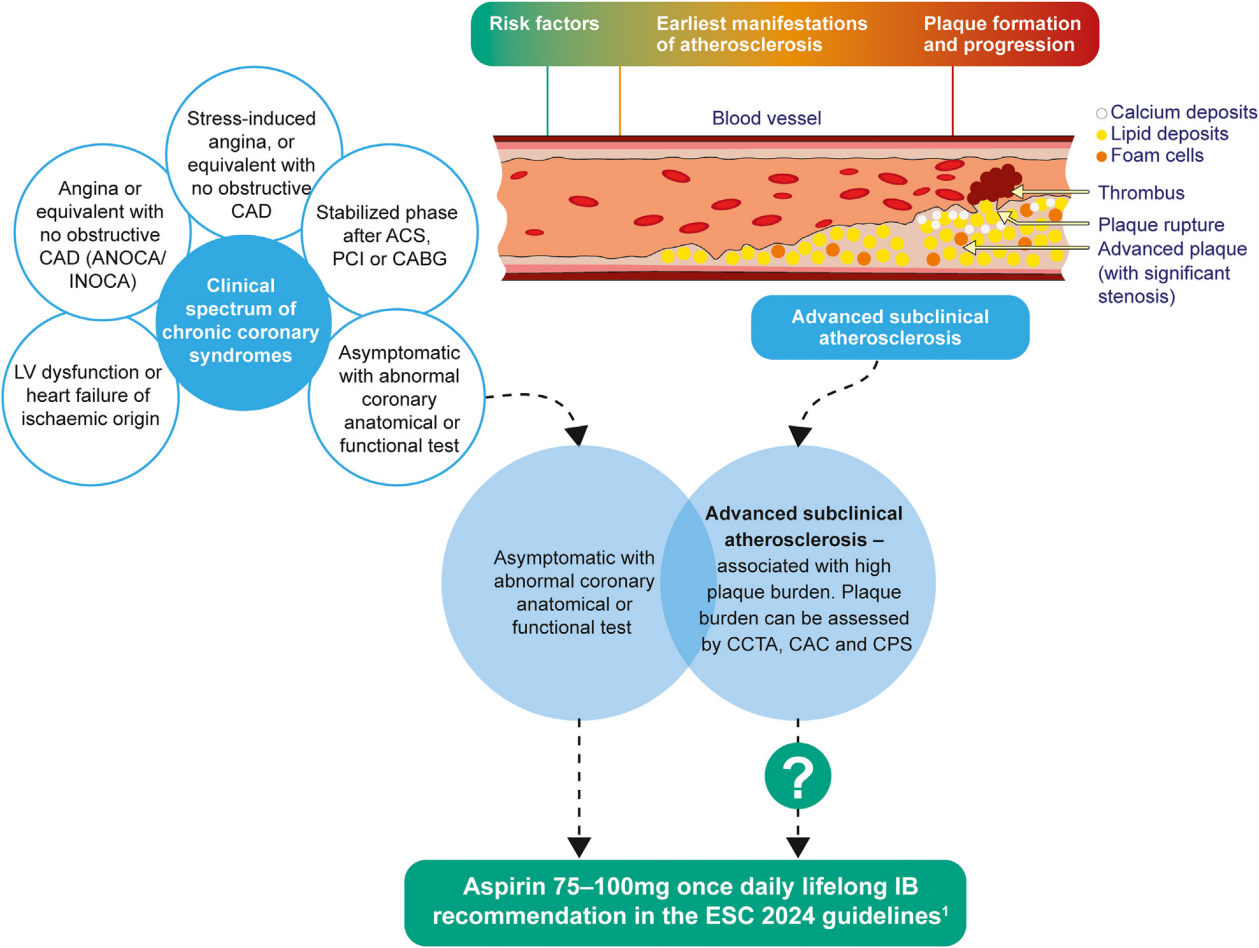
Correlation Between Asymptomatic Chronic Coronary Syndrome, Advanced Subclinical Atherosclerosis, and Low-Dose Aspirin ACS = acute coronary syndrome; ANOCA = angina with no obstructive CAD; CABG = coronary artery bypass graft; CAC = coronary artery calcium; CAD = coronary artery disease; CCS = chronic coronary syndrome; CCTA = coronary computed tomography angiography; CPS = carotid plaque ultrasound; ESC = European Society of Cardiology; INOCA = ischemia with no obstructive CAD; LV = left ventricular; PCI = percutaneous coronary intervention.

As it is almost never recommended to actively search for obstructive CAD in asymptomatic patients, we propose to consider patients with advanced subclinical atherosclerosis, determined by any proven method, as having CCS, even if the degree of coronary stenosis has not been evaluated. Moreover, since low-dose aspirin is associated with a risk of bleeding, we recommend weighing the benefits and risks of low-dose aspirin therapy and prescribing low-dose aspirin only for patients aged <70 years who are projected to obtain a clear net clinical benefit from preventive therapy. As such, patients with no detectable atherosclerosis should rarely if ever take aspirin.

To some, our recommendations may not seem new or may seem obvious. But for too long, guidelines have been arbitrarily placed into 2 distinct buckets—primary and secondary prevention. The CCS concept is consistent with our approach to coronary risk as a continuum, best expressed by the degree of atherosclerotic plaque (Figure 1). The concept of advanced subclinical atherosclerosis can remove some of the ambiguity around the appropriate use of aspirin, which has both benefits and harms and is probably best thought of as removed from the strict primary vs secondary prevention dichotomy.

In conclusion, the modern understanding of coronary risk has led to the merging of the concepts of advanced subclinical atherosclerosis and asymptomatic obstructive CAD. As such, we believe that advanced subclinical atherosclerosis is part of the asymptomatic CCS as described by the ESC guidelines. Therefore, the new ESC recommendations for the use of lifelong low-dose aspirin in patients with asymptomatic CCS but with evidence of CAD on imaging should probably apply to many or even most patients with advanced subclinical atherosclerosis. In fact, modeling studies suggest that the tipping point for net aspirin benefit is CAC >100 or moderate plaque on carotid ultrasound (carotid plaque score of 2 or greater) in the absence of bleeding risk factors. Future prevention and American College of Cardiology/American Heart Association CCS guidelines should consider these criteria for more specific and individualized aspirin use in practice.

## Funding support and author disclosures

Dr Blaha is a member of advisory boards: Novo Nordisk, Novartis, Bayer, Eli Lilly, AstraZeneca, Boehringer Ingelheim, Idorsia, Genentech, Agepha, Vectura, and New Amsterdam. Received grants from 10.13039/100000002NIH, 10.13039/100000038FDA, 10.13039/100000968AHA, Novo Nordisk, 10.13039/100004326Bayer, 10.13039/100002429Amgen. Dr Santilli is a member of a Bayer AG global advisory board that convenes to discuss risk assessment and preventive pharmacotherapy in primary and secondary prevention. Dr Sibbing receives speaker fees and fees from advisory board activities from Bayer, Sanofi, and Daiichi Sankyo. Dr Cobo is a member of a Bayer AG global advisory board that convenes to discuss risk assessment and preventive pharmacotherapy in primary and secondary prevention. Received speaker honoraria from Novartis, Novo Nordisk, AstraZeneca, and Servier. Dr Abdelhamid is a member of a Bayer AG global advisory board that convenes to discuss risk assessment and preventive pharmacotherapy in primary and secondary prevention. Received speaker honoraria from Bayer, Novartis, AstraZeneca, and Pfizer. Dr Albrecht is an employee of Bayer. Dr Li is an employee of Bayer.
